# Supplementary material for: Comparative Study of the Lipophilicity of Selected Anti-Androgenic and Blood Uric Acid Lowering Compounds
Source: Molecules. 2022 Dec 25;28(1):166. doi: 10.3390/molecules28010166 (PMC9821840; doi:10.3390/molecules28010166)
Supplement: Supplementary file 1 [file molecules-28-00166-s001.zip › molecules-2102356-supplementary.pdf]

Table S1. Data for linear correlation (Equation 1) between R_M values and the content of organic modifier in the mobile phase for allopurinol. Where: correlation coefficient (R^2), standard error of estimation (SEE); F-factor; significance level (p), volume fraction of organic modifier in mobile phase (φ).

			**Ethanol – water (v/v)**				
**Stationary phase**	**R_{MW}±SE**	**b±SE**	**R^2**	**SEE**	**F**	**p**	**φ**
RP18F_{254}	0.2164±0.0923	-1.7360±0.2176	95.50	0.0688	63.61	0.0041	0.20 – 0.80
RP18WF_{254}	0.2321±0.0541	-1.8966±0.1125	98.61	0.0471	284.21	0.0001	0.20 – 0.70
RP2F_{254}	0.4000±0.1346	-2.2336±0.2500	94.10	0.1323	79.81	0.0003	0.20 – 0.80
			**Acetonitrile – water (v/v)**				
**Stationary phase**	**R_{MW}±SE**	**b±SE**	**R^2**	**SEE**	**F**	**p**	**φ**
RP18F_{254}	-0.0737±0.0706	-1.0200±0.1185	92.51	0.0768	74.10	0.0001	0.20 – 0.90
RP18WF_{254}	-0.1744±0.0499	-0.8414±0.0927	94.28	0.0490	82.41	0.0003	0.20 – 0.80
RP2F_{254}	0.2783±0.0856	-2.8830±0.2330	98.71	0.0521	153.23	0.0065	0.20 – 0.50
			**Propan-2-ol – water (v/v)**				
**Stationary phase**	**R_{MW}±SE**	**b±SE**	**R^2**	**SEE**	**F**	**p**	**φ**
RP18F_{254}	0.0399±0.0767	-1.4000±0.1425	95.08	0.0754	96.56	0.0002	0.20 – 0.80
RP18WF_{254}	-0.0722±0.1766	-1.2343±0.3670	73.87	0.1535	11.31	0.0282	0.20 – 0.70
RP2F_{254}	-0.0849±0.1014	-1.2114±0.2106	89.21	0.0881	33.07	0.0045	0.20 – 0.70

Table S2. Data for linear correlation (Equation 1) between R_M values and the content of organic modifier in the mobile phase for oxypurinol. Where: correlation coefficient (R^2), standard error of estimation (SEE); F-factor; significance level (p), volume fraction of organic modifier in mobile phase (φ).

			**Ethanol – water (v/v)**				
**Stationary phase**	**R_{MW}±SE**	**b±SE**	**R^2**	**SEE**	**F**	**p**	**φ**
RP18F_{254}	0.1808±0.0699	-2.0100±0.1647	98.02	0.0521	148.92	0.0012	0.20 – 0.60
RP18WF_{254}	-0.0346±0.2414	-2.4520±0.5016	85.66	0.2098	23.89	0.0081	0.20 – 0.70
RP2F_{254}	-0.5431±0.1278	-1.2830±0.2459	90.07	0.0777	27.23	0.0137	0.30 – 0.70
			**Acetonitrile – water (v/v)**				
**Stationary phase**	**R_{MW}±SE**	**b±SE**	**R^2**	**SEE**	**F**	**p**	**φ**
RP18F_{254}	0.3765±0.2038	-1.6590±0.2853	91.85	0.0902	33.81	0.0101	0.50 – 0.90
RP18WF_{254}	-0.2654±0.0719	-0.9406±0.1493	90.84	0.0625	39.69	0.0032	0.20 – 0.70
RP2F_{254}	0.7764±0.2288	-4.2400±0.5394	95.37	0.1706	61.79	0.0043	0.20 – 0.60
			**Propan-2-ol – water (v/v)**				
**Stationary phase**	**R_{MW}±SE**	**b±SE**	**R^2**	**SEE**	**F**	**p**	**φ**
RP18F_{254}	-0.3749±0.1099	-0.7752±0.1844	74.65	0.1195	17.67	0.0057	0.20 – 0.90
RP18WF_{254}	-0.5789±0.0057	-0.3514±0.0099	99.92	0.0021	1260.75	0.0179	0.40 – 0.70
RP2F_{254}	-0.1945±0.0862	-1.1849±0.1451	97.09	0.0429	66.64	0.0147	0.40 – 0.80

Table S3. Data for linear correlation (Equation 1) between R_M values and the content of organic modifier in the mobile phase for febuxostat. Where: correlation coefficient (R^2), standard error of estimation (SEE); F-factor; significance level (p), volume fraction of organic modifier in mobile phase (φ).

**Ethanol – water (v/v)**							
**Stationary phase**	**R_{MW}±SE**	**b±SE**	**R^2**	**SEE**	**F**	**p**	**φ**
$RP18F_{254}$	2.7106±0.2849	-4.4626±0.4947	95.31	0.2069	81.37	0.0008	0.30 – 0.80
$RP18WF_{254}$	2.0687±0.3224	-3.9743±0.5986	89.81	0.3167	44.08	0.0012	0.20 – 0.80
$RP2F_{254}$	1.9788±0.3416	-3.9960±0.5931	91.90	0.2481	45.39	0.0025	0.30 – 0.80
**Acetonitrile – water (v/v)**							
**Stationary phase**	**R_{MW}±SE**	**b±SE**	**R^2**	**SEE**	**F**	**p**	**φ**
$RP18F_{254}$	1.9061±0.1300	-2.7420±0.1934	98.05	0.0809	201.05	0.0001	0.40 – 0.90
$RP18WF_{254}$	1.5489±0.0517	-2.7251±0.0868	99.39	0.0563	985.48	0.0000	0.20 – 0.90
$RP2F_{254}$	1.5648±0.1540	-3.0405±0.2584	95.85	0.1675	138.45	0.0000	0.20 – 0.90
**Propan-2-ol – water (v/v)**							
**Stationary phase**	**R_{MW}±SE**	**b±SE**	**R^2**	**SEE**	**F**	**p**	**φ**
$RP18F_{254}$	1.9112±0.2491	-3.2326±0.4182	90.88	0.2710	59.76	0.0002	0.20 – 0.90
$RP18WF_{254}$	1.2259±0.1424	-2.4143±0.2390	94.45	0.1549	102.08	0.0001	0.20 – 0.90
$RP2F_{254}$	1.2524±0.1654	-2.7598±0.2776	94.27	0.1799	98.79	0.0001	0.20 – 0.90

Table S4. Data for linear correlation (Equation 1) between R_M values and the content of organic modifier in the mobile phase for abiraterone. Where: correlation coefficient (R^2), standard error of estimation (SEE); F-factor; significance level (p), volume fraction of organic modifier in mobile phase (φ).

**Ethanol – water (v/v)**							
**Stationary phase**	**R_{MW}±SE**	**b±SE**	**R^2**	**SEE**	**F**	**p**	**φ**
$RP18F_{254}$	4.5660±0.7096	-4.7950±0.9359	92.92	0.2093	26.25	0.0360	0.60 – 0.90
$RP18WF_{254}$	3.8765±0.3779	-4.4090±0.4984	97.51	0.1114	78.27	0.0125	0.60 – 0.90
$RP2F_{254}$	4.3012±0.5620	-5.4360±0.7869	94.08	0.2488	47.72	0.0062	0.50 – 0.90
**Acetonitrile – water (v/v)**							
**Stationary phase**	**R_{MW}±SE**	**b±SE**	**R^2**	**SEE**	**F**	**p**	**φ**
$RP18F_{254}$	1.8373±0.0580	-0.6650±0.0722	98.84	0.0102	84.91	0.0688	0.70 – 0.90
$RP18WF_{254}$	2.4400±0.0824	-2.0930±0.1087	99.46	0.0243	370.52	0.0027	0.40 – 0.90
$RP2F_{254}$	2.7366±0.2768	-3.1920±0.3876	95.76	0.1226	67.82	0.0037	0.50 – 0.90
**Propan-2-ol – water (v/v)**							
**Stationary phase**	**R_{MW}±SE**	**b±SE**	**R^2**	**SEE**	**F**	**p**	**φ**
$RP18F_{254}$	3.0060±0.2713	-3.6220±0.3800	96.80	0.1202	90.87	0.0024	0.50 – 0.90
$RP18WF_{254}$	2.0141±0.3497	-2.4610±0.4897	89.38	0.1549	25.25	0.0152	0.50 – 0.90
$RP2F_{254}$	2.5179±0.2130	-3.6837±0.3170	97.12	0.1326	135.05	0.0003	0.40 – 0.90

Table S5. Data for linear correlation (Equation 1) between R_M values and the content of organic modifier in the mobile phase for bicalutamide. Where: correlation coefficient (R^2), standard error of estimation (SEE); F-factor; significance level (p), volume fraction of organic modifier in mobile phase (φ).

Stationary phase	R_{MW}±SE	b±SE	R^2	SEE	F	p	φ
**Ethanol – water (v/v)**							
$RP18F_{254}$	3.1861±0.7150	-5.1317±1.0638	85.33	0.4450	23.27	0.0085	0.40 – 0.90
$RP18WF_{254}$	2.8711±0.2530	-4.7611±0.3765	97.56	0.1575	159.94	0.0002	0.40 – 0.90
$RP2F_{254}$	2.8044±0.2780	-4.5411±0.4136	96.79	0.1730	120.55	0.0004	0.40 – 0.90
**Acetonitrile – water (v/v)**							
**Stationary phase**	**R_{MW}±SE**	**b±SE**	**R^2**	**SEE**	**F**	**p**	**φ**
$RP18F_{254}$	4.0113±0.3574	-6.3340±0.5318	97.26	0.2225	141.86	0.0003	0.40 – 0.90
$RP18WF_{254}$	2.8946±0.2591	-4.9186±0.3855	97.60	0.1613	162.77	0.0002	0.40 – 0.90
$RP2F_{254}$	3.0843±0.1717	-5.1040±0.2555	99.01	0.1069	399.13	0.0000	0.40 – 0.90
**Propan-2-ol – water (v/v)**							
**Stationary phase**	**R_{MW}±SE**	**b±SE**	**R^2**	**SEE**	**F**	**p**	**φ**
$RP18F_{254}$	2.7550±0.4628	-5.3240±0.7507	94.37	0.2374	50.29	0.0058	0.40 – 0.80
$RP18WF_{254}$	1.4439±0.4882	-2.9134±0.7265	80.08	0.3039	16.08	0.0160	0.40 – 0.90
$RP2F_{254}$	1.9488±0.1060	-3.9310±0.1720	99.43	0.0544	522.13	0.0002	0.40 – 0.80

Table S6. Data for linear correlation (Equation 1) between R_M values and the content of organic modifier in the mobile phase for flutamide. Where: correlation coefficient (R^2), standard error of estimation (SEE); F-factor; significance level (p), volume fraction of organic modifier in mobile phase (φ).

Stationary phase	R_{MW}±SE	b±SE	R^2	SEE	F	p	φ
**Ethanol – water (v/v)**							
$RP18F_{254}$	3.1037±0.2366	-4.4163±0.3520	97.52	0.1473	157.37	0.0002	0.40 – 0.90
$RP18WF_{254}$	2.9650±0.2815	-4.5594±0.4189	96.73	0.1753	118.45	0.0004	0.40 – 0.90
$RP2F_{254}$	2.9756±0.3050	-4.5286±0.4539	96.14	0.1899	99.55	0.0006	0.40 – 0.90
**Acetonitrile – water (v/v)**							
**Stationary phase**	**R_{MW}±SE**	**b±SE**	**R^2**	**SEE**	**F**	**p**	**φ**
$RP18F_{254}$	3.3395±0.2534	-4.8223±0.3771	97.61	0.1577	163.57	0.0002	0.40 – 0.90
$RP18WF_{254}$	2.7053±0.1620	-4.4240±0.2410	98.83	0.1008	336.99	0.0001	0.40 – 0.90
$RP2F_{254}$	2.6436±0.1123	-4.2146±0.1671	99.37	0.0699	636.08	0.0000	0.40 – 0.90
**Propan-2-ol – water (v/v)**							
**Stationary phase**	**R_{MW}±SE**	**b±SE**	**R^2**	**SEE**	**F**	**p**	**φ**
$RP18F_{254}$	2.2432±0.2168	-3.8526±0.3226	97.27	0.1350	142.61	0.0003	0.40 – 0.90
$RP18WF_{254}$	1.7854±0.3287	-3.1654±0.4892	91.28	0.2046	41.88	0.0029	0.40 – 0.90
$RP2F_{254}$	2.4137±0.2010	-4.4551±0.2990	98.23	0.1251	221.94	0.0001	0.40 – 0.90

Table S7. Data for linear correlation (Equation 1) between R_M values and the content of organic modifier in the mobile phase for nilutamide. Where: correlation coefficient (R^2), standard error of estimation (SEE); F-factor; significance level (p), volume fraction of organic modifier in mobile phase (φ).

Stationary phase	R_{MW}±SE	b±SE	R^2	SEE	F	p	φ
**Ethanol – water (v/v)**							
$RP18F_{254}$	2.8873±0.2044	-4.3151±0.3041	98.05	0.1272	201.29	0.0001	0.40 – 0.90
$RP18WF_{254}$	2.3245±0.1433	-3.6883±0.2132	98.68	0.0892	299.35	0.0001	0.40 – 0.90
$RP2F_{254}$	3.0454±0.2606	-4.2900±0.3649	97.87	0.1154	138.19	0.0013	0.50 – 0.90
**Acetonitrile – water (v/v)**							
**Stationary phase**	**R_{MW}±SE**	**b±SE**	**R^2**	**SEE**	**F**	**p**	**φ**
$RP18F_{254}$	3.1543±0.2336	-5.1300±0.3476	98.20	0.1454	217.84	0.0001	0.40 – 0.90
$RP18WF_{254}$	2.3260±0.2136	-4.0246±0.3178	97.57	0.1329	160.41	0.0002	0.40 – 0.90
$RP2F_{254}$	2.7675±0.1070	-4.7374±0.1592	99.55	0.0666	885.30	0.0000	0.40 – 0.90
**Propan-2-ol – water (v/v)**							
**Stationary phase**	**R_{MW}±SE**	**b±SE**	**R^2**	**SEE**	**F**	**p**	**φ**
$RP18F_{254}$	1.9313±0.2861	-3.5194±0.4257	94.47	0.1781	68.33	0.0012	0.40 – 0.90
$RP18WF_{254}$	1.4803±0.3582	-2.6889±0.5330	86.42	0.2230	25.45	0.0073	0.40 – 0.90
$RP2F_{254}$	2.1987±0.1545	-4.2757±0.2299	98.86	0.0962	345.94	0.0000	0.40 – 0.90

Table S8. Data for linear correlation (Equation 1) between R_M values and the content of organic modifier in the mobile phase for leflunomide. Where: correlation coefficient (R^2), standard error of estimation (SEE); F-factor; significance level (p), volume fraction of organic modifier in mobile phase (φ).

Stationary phase	R_{MW}±SE	b±SE	R^2	SEE	F	p	φ
**Ethanol – water (v/v)**							
$RP18F_{254}$	3.5015±0.3566	-4.9571±0.5306	95.62	0.2220	87.29	0.0007	0.40 – 0.90
$RP18WF_{254}$	2.8223±0.1406	-4.4094±0.2092	99.11	0.0875	443.94	0.0000	0.40 – 0.90
$RP2F_{254}$	3.0335±0.2793	-4.5871±0.4156	96.82	0.1738	121.83	0.0004	0.40 – 0.90
**Acetonitrile – water (v/v)**							
**Stationary phase**	**R_{MW}±SE**	**b±SE**	**R^2**	**SEE**	**F**	**p**	**φ**
$RP18F_{254}$	3.0430±0.2314	-4.4600±0.3444	97.67	0.1441	167.74	0.0002	0.40 – 0.90
$RP18WF_{254}$	2.6675±0.1872	-4.3820±0.2785	98.41	0.1165	247.47	0.0001	0.40 – 0.90
$RP2F_{254}$	2.7559±0.0730	-4.3989±0.1086	99.76	0.0454	1641.20	0.0000	0.40 – 0.90
**Propan-2-ol – water (v/v)**							
**Stationary phase**	**R_{MW}±SE**	**b±SE**	**R^2**	**SEE**	**F**	**p**	**φ**
$RP18F_{254}$	2.3386±0.2911	-4.0286±0.4332	95.58	0.1812	86.47	0.0007	0.40 – 0.90
$RP18WF_{254}$	1.8840±0.4102	-3.3660±0.6104	88.38	0.2553	30.41	0.0053	0.40 – 0.90
$RP2F_{254}$	2.5391±0.2044	-4.6023±0.3042	98.28	0.1273	228.89	0.0001	0.40 – 0.90

Table S9. Data for linear correlation (Equation 1) between R_M values and the content of organic modifier in the mobile phase for teriflunomide. Where: correlation coefficient (R^2), standard error of estimation (SEE); F-factor; significance level (p), volume fraction of organic modifier in mobile phase (φ).

Ethanol – water (v/v)							
**Stationary phase**	**R_{MW}±SE**	**b±SE**	**R^2**	**SEE**	**F**	**p**	**φ**
RP18F254	1.9800±0.5223	-3.9594±0.7771	86.65	0.3251	25.96	0.0070	0.40 – 0.90
RP18WF254	1.2736±0.1409	-3.1334±0.2096	98.24	0.0877	223.51	0.0001	0.40 – 0.90
RP2F254	1.3228±0.1929	-3.0120±0.2871	96.49	0.1201	110.07	0.0005	0.40 – 0.90
**Acetonitrile – water (v/v)**							
**Stationary phase**	**R_{MW}±SE**	**b±SE**	**R^2**	**SEE**	**F**	**p**	**φ**
RP18F254	1.3186±0.0596	-2.8164±0.1027	99.87	0.0222	752.27	0.0232	0.40 – 0.70
RP18WF254	1.0250±0.2466	-2.5449±0.3669	92.32	0.1535	48.10	0.0023	0.40 – 0.90
RP2F254	0.9696±0.1000	-2.7820±0.1783	99.18	0.0399	243.47	0.0041	0.40 – 0.70
**Propan-2-ol – water (v/v)**							
**Stationary phase**	**R_{MW}±SE**	**b±SE**	**R^2**	**SEE**	**F**	**p**	**φ**
RP18F254	2.0438±0.3051	-4.600±0.4950	96.64	0.1565	86.36	0.0026	0.40 – 0.80
RP18WF254	1.8615±0.2917	-4.6750±0.5197	97.59	0.1162	80.91	0.0121	0.40 – 0.70
RP2F254	1.0884±0.2339	-3.0920±0.3795	95.68	0.1200	66.38	0.0039	0.40 – 0.80

Table S10. Data for linear correlation (Equation 1) between R_M values and the content of organic modifier in the mobile phase for ailanthone. Where: correlation coefficient (R^2), standard error of estimation (SEE); F-factor; significance level (p), volume fraction of organic modifier in mobile phase (φ).

Ethanol – water (v/v)							
**Stationary phase**	**R_{MW}±SE**	**b±SE**	**R^2**	**SEE**	**F**	**p**	**φ**
RP18F254	0.6137±0.4460	-2.3063±0.6637	75.12	0.2776	12.08	0.0255	0.40 – 0.90
RP18WF254	0.3212±0.1124	-1.1837±0.1673	92.60	0.0700	50.06	0.0021	0.40 – 0.90
RP2F254	0.8299±0.3079	-2.7543±0.4581	90.04	0.1916	36.15	0.0039	0.40 – 0.90
**Acetonitrile – water (v/v)**							
**Stationary phase**	**R_{MW}±SE**	**b±SE**	**R^2**	**SEE**	**F**	**p**	**φ**
RP18F254	0.9749±0.0122	-2.3693±0.0210	99.99	0.0045	12690.30	0.0057	0.40 – 0.70
RP18WF254	1.2586±0.3190	-2.9270±0.5683	92.99	0.1271	26.53	0.0357	0.40 – 0.70
RP2F254	0.4203±0.1201	-1.3760±0.2139	95.39	0.0478	41.37	0.0233	0.40 – 0.70
**Propan-2-ol – water (v/v)**							
**Stationary phase**	**R_{MW}±SE**	**b±SE**	**R^2**	**SEE**	**F**	**p**	**φ**
RP18F254	1.7092±0.5273	-4.1680±0.7995	93.15	0.1788	27.18	0.0349	0.50 – 0.80
RP18WF254	0.2488±0.0566	-1.2410±0.1009	98.69	0.0226	151.18	0.0065	0.40 – 0.70
RP2F254	0.7606±0.3251	-3.036±0.5274	91.70	0.1668	33.13	0.0104	0.40 – 0.80

Supplementary Materials: The following materials can be downloaded at: www.mdpi.com/xxx/s1, Table S1: Data for linear correlation (Equation 1) between R_M values and the content of organic modifier in the mobile phase for allopurinol. Where: correlation coefficient (R^2), standard error of estimation (SEE); F-factor; significance level (p), volume fraction of organic modifier in mobile phase (φ); Table S2: Data for linear correlation (Equation 1) between R_M values and the content of organic modifier in the mobile phase for oxypurinol. Where: correlation coefficient (R^2), standard error of estimation (SEE); F-factor; significance level (p), volume fraction of organic modifier in mobile phase (φ); Table S3: Data for linear correlation (Equation 1) between R_M values and the content of organic modifier in the mobile phase for febuxostat. Where: correlation coefficient (R^2), standard error of estimation (SEE); F-factor; significance level (p), volume fraction of organic modifier in mobile phase (φ); Table S4: Data for linear correlation (Equation 1) between R_M values and the content of organic modifier in the mobile phase for abiraterone. Where: correlation coefficient (R^2), standard error of estimation (SEE); F-factor; significance level (p), volume fraction of organic modifier in mobile phase (φ); Table S5: Data for linear correlation (Equation 1) between R_M values and the content of organic modifier in the mobile phase for bicalutamide. Where: correlation coefficient (R^2), standard error of estimation (SEE); F-factor; significance level (p), volume fraction of organic modifier in mobile phase (φ); Table S6: Data for linear correlation (Equation 1) between R_M values and the content of organic modifier in the mobile phase for flutamide. Where: correlation coefficient (R^2), standard error of estimation (SEE); F-factor; significance level (p), volume fraction of organic modifier in mobile phase (φ); Table S7: Data for linear correlation (Equation 1) between R_M values and the content of organic modifier in the mobile phase for nilutamide. Where: correlation coefficient (R^2), standard error of estimation (SEE); F-factor; significance level (p), volume fraction of organic modifier in mobile phase (φ); Table S8: Data for linear correlation (Equation 1) between R_M values and the content of organic modifier in the mobile phase for leflunomide. Where: correlation coefficient (R^2), standard error of estimation (SEE); F-factor; significance level (p), volume fraction of organic modifier in mobile phase (φ); Table S9: Data for linear correlation (Equation 1) between R_M values and the content of organic modifier in the mobile phase for teriflunomide. Where: correlation coefficient (R^2), standard error of estimation (SEE); F-factor; significance level (p), volume fraction of organic modifier in mobile phase (φ); Table S10: Data for linear correlation (Equation 1) between R_M values and the content of organic modifier in the mobile phase for ailanthone. Where: correlation coefficient (R^2), standard error of estimation (SEE); F-factor; significance level (p), volume fraction of organic modifier in mobile phase (φ).
